# Structure and ligand binding of the SAM-V riboswitch

**DOI:** 10.1093/nar/gky520

**Published:** 2018-06-21

**Authors:** Lin Huang, David M J Lilley

**Affiliations:** Cancer Research UK Nucleic Acid Structure Research Group, MSI/WTB Complex, The University of Dundee, Dow Street, Dundee DD1 5EH, UK

## Abstract

SAM-V is one of the class of riboswitches that bind *S*-adenosylmethione, regulating gene expression by controlling translation. We have solved the crystal structure of the *met*Y SAM-V riboswitch bound to its SAM ligand at 2.5 Å resolution. The RNA folds as an H-type pseudoknot, with a major-groove triple helix in which resides the SAM ligand binding site. The bound SAM adopts an elongated conformation aligned with the axis of the triple helix, and is held at either end by hydrogen bonding to the adenine and the amino acid moieties. The central sulfonium cation makes electrostatic interactions with an U:A.U base triple, so conferring specificity. We propose a model in which SAM binding leads to association of the triplex third strand that stabilizes a short helix and occludes the ribosome binding site. Thus the new structure explains both ligand specificity and the mechanism of genetic control.

## INTRODUCTION

Riboswitches are elements that occur in untranslated regions of mainly bacterial mRNA, that fold to bind ligands with great selectivity leading to the control of gene expression (1–3). In general the ligands are small molecules that are related to the metabolites that are processed in some manner (e.g. chemically converted or transported) by the gene products. High specificity is an important property of riboswitches; they must bind their ligand and exclude metabolites of similar structure. In most cases ligand binding changes the conformation of the riboswitch such that either transcriptional termination is modulated (4), or the accessibility of a ribosome binding site is altered to modulate translation (5). Ligand binding can result in levels of gene expression that are decreased (OFF switch) or increased (ON switch). Riboswitches are known that respond to a variety of metabolites including co-enzymes, nucleobases, amino acids and even single ions, and in some cases different classes of riboswitch exist that respond to the same metabolite, with different structures and sometimes different regulatory mechanisms.

The *S*-adenosylmethionine (SAM) riboswitches provide an example of a widespread family containing a number of different classes. As a group they bind SAM with different RNA folds, creating different ligand binding sites and using different mechanisms to regulate gene expression. The first to be described was the S-box or SAM-I riboswitch (6,7), found as multiple examples in many gram-positive bacterial species such as *Bacillus subtilis*. This riboswitch controls a number of genes involved in sulfur metabolism, acting as a transcriptional OFF switch. The structure of the SAM-I riboswitch (8) showed it to be based on a four-way junction with a k-turn mediating a loop–receptor interaction to create the SAM binding site. Subsequently a number of other classes of SAM-binding riboswitches have been discovered, including the SAM-II riboswitches in α-proteobacteria (9) and SAM-III riboswitches in Lactobacillales (10). Structure determination of the SAM-II (11) and SAM-III riboswitches (12) showed that they were completely different both from the SAM-I riboswitch (8) and each other. The SAM-III riboswitch is based on a three-way helical junction, while the SAM-II riboswitch is organized as a pseudoknot. This difference exemplifies the observation that small, autonomously-folding RNA species are frequently based around either helical junctions or pseudoknots. The conformation of SAM and the manner of its binding also differs between these three classes of riboswitch, although in each the SAM adenine moiety is base paired to nucleobases from the RNA.

Breaker and co-workers (13) described a new class of SAM riboswitches called SAM-V, that was found by bioinformatic analysis of intergenic regions of marine α-protobacteria and bacteroidetes. Poiata *et al*. (13) noted that the secondary structure of the new riboswitch class was closely similar to the SAM-II riboswitch and predicted that they would adopt similar structures. However, they are phylogenetically distinct, and importantly SAM-V riboswitches are translational whereas the majority of SAM-II are transcriptional riboswitches. We therefore set out to crystallize the SAM-V riboswitch and solve its three-dimensional structure by X-ray crystallography. On the basis of this we deduce how ligand specificity is ensured, and present a model for the action of the RNA as a translational OFF switch.

## MATERIALS AND METHODS

### RNA synthesis

The RNA sequences used in this study are shown in Supplementary Table S1. RNA oligonucleotides were synthesized using ***t***-BDMS phosphoramidite chemistry (14) as described in Wilson *et al*. (15), implemented on an Applied Biosystems 394DNA/RNA synthesizer. Oligoribonucleotides containing inosine (Glen Research), purine (ChemGenes) and 4-thiouridine (ChemGenes) were deprotected in a 25% ethanol/ammonia solution for 4 h at 20°C, and evaporated to dryness. Oligoribonucleotides containing isocytidine (ChemGenes) were further deprotected for 2 h at 65°C. Oligoribonucleotides containing 5-bromocytidine (ChemGenes) were deprotected 36 h at 20°C. All oligoribonucleotides were redissolved in 100 μl of anhydrous DMSO and 125 μl triethylamine trihydrofluoride (Sigma-Aldrich) to remove t-BDMS groups, and agitated at 65°C in the dark for 2.5 h. After cooling on ice for 10 min, the RNA was precipitated with 1 ml of butanol, washed twice with 70% ethanol and suspended in double-distilled water.

RNA was further purified by gel electrophoresis in polyacrylamide under denaturing conditions in the presence of 7 M urea. The full-length RNA product was visualized by UV shadowing. The band was excised and electroeluted using an Elutrap Electroelution System (GE Healthcare) into 45 mM Tris-borate (pH 8.5), 5 mM ethylenediaminetetraacetic acid buffer for 8 h at 200 V at 4°C. The RNA was precipitated with ethanol, washed once with 70% ethanol and dissolved in double-distilled water.

### Crystallization, structure determination and refinement

A solution of 1 mM *met*Y SAM-V riboswitch RNA (53 nt, Supplementary Table S1) in 5 mM HEPES (pH 7.6), 100 mM KCl was heated to 95°C for 1 min. The solution was slowly cooled to 20°C and MgCl_2_ added to a final concentration of 2 mM. SAM (Sigma-Aldrich) was added to a final concentration of 5 mM. Crystals were grown by sitting drop vapor diffusion at 7°C using drops prepared by mixing 0.3 μl of the RNA–ligand complex with 0.3 μl of a reservoir solution comprising 50 mM Bis-Tris (pH 7.0), 200 mM KCl, 10 mM CaCl_2_ dihydrate and 40% v/vPEG 400 (HELIX26) (16) using a Mosquito robot (TTP LabTech). Crystals (of approximate dimensions 50 × 50 × 50 μm^3^) with space group P4_1_22 appeared after 7 days. The crystals were flash frozen by mounting in nylon loops and plunging into liquid nitrogen.

Diffraction data were collected on beamline I04-1 and I03 of Diamond Light Source (Harwell, UK), under proposal number MX14980. Data were processed by XIA2 (17) The resolution cutoff for the data was determined by examining by CC_1/2_ and density map as described previously (18). Initial phase information was acquired from the SAD data by locating the bromine atom with Autosol in the PHENIX suite. Models were adjusted manually using Coot (19) and subjected to several rounds of adjustment and optimization using Coot, phenix.refine and PDB_REDO (20). Model geometry and the fit to the electron density maps were monitored with MOLPROBITY (21) and the validation tools in Coot. The unbiased electron density maps were generated through Br-SAD phasing and density modification by Phenix AutoSol.

### Isothermal titration calorimetry

The titrations were performed at 298 K using an ITC-200 microcalorimeter (GE). RNA solutions (15–20 μM) were prepared by diluting concentrated stocks into the binding buffer containing 40 mM HEPES (pH 7.2), 100 mM KCl, 10 mM MgCl_2_. SAM and SAH was prepared in the same binding buffer with a concentration of 200–250 μM. The sample cell was filled with 200 μl of RNA. Solutions were degassed for 2–5 min before loading. The sample cell was filled with 200 μl of RNA. SAM or SAH was injected in a volume of 0.4 μl for the first injection and 2 μl for the next 19 injections using a computer-controlled 40 μl microsyringe with an injection interval of 120 s. Titration of ligands into the binding buffer or titration of the binding buffer into the RNA solution resulted in negligible evolution of heat. Integrated heat data were analyzed using a one-set-of-sites model in MicroCal Origin following the manufacturer's instructions. The first data point was excluded in analysis. The binding parameters enthalpy Δ*H* (cal mol^−1^), association constant *K* (M^−1^) and *n* (bound ligands per RNA) were variables in the fit. The binding free energy Δ*G* and reaction entropy Δ*S* were calculated using the relationships Δ*G*  =  −*RT* ln *K*, where *R* =  1.987 cal mol^−1^ K^−1^, *T* = 298 K and Δ*G* = Δ*H* − *TΔS*. The dissociation constant *K*_d_ was calculated as 1/*K*.

### Sequence alignment and analysis

SAM V sequences were taken from supplemental data of Meyer *et al*. (22) and SAM-II sequences were taken from Rfam under accession RF00521. The sequences were manually realigned against the crystal structure using Jalview (23). All sequence composition and covariation analysis was calculated by Jalview.

## RESULTS

### Calorimetric analysis of ligand binding by the SAM-V riboswitch

**Figure 1.**
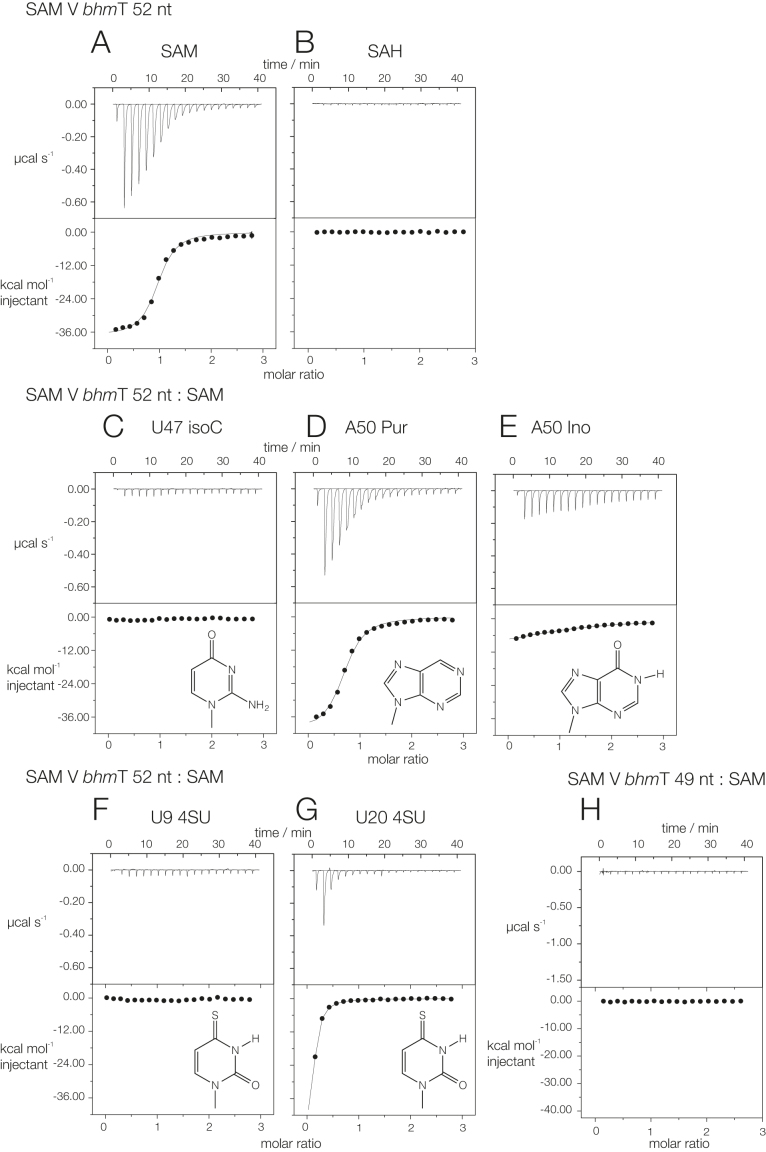
Isothermal titration calorimetric measurement of ligand binding to the *bhm*T SAM-V riboswitch and variants. A solution of ligand (SAM except for part **B**) was titrated into a SAM-V riboswitch solution, and the heat evolved was measured as the power required to maintain zero temperature difference with a reference cell. Integration over time gives the heat required to maintain thermal equilibrium between cells. In each case the upper panel shows the raw data for sequential injections of 2 μl volumes (following an initial injection of 0.4 μl) of a 200 μM solution of SAM into 200 μl of a 15 μM RNA solution in 40 mM HEPES (pH 7.2), 100 mM KCl, 10 mM MgCl_2_. This represents the differential of the total heat (i.e. enthalpy Δ*H*° under conditions of constant pressure) for each SAM concentration. The lower panels present the integrated heat data fitted (where possible) to a single-site binding model. The thermodynamic parameters calculated are summarized in Supplementary Table S2. The ITC analysis was performed for the SAM V riboswitch in which the riboswitch sequence was modified as follows: (**A**) SAM titrated into SAM-52 (i.e. *bhm*T SAM-V riboswitch of 52 nt, see Supplementary Table S1). (B) SAH titrated into SAM-52. (**C–E**). SAM titrated into SAM-52 with substitutions U47Isocytidine, A50Purine and A50Inosine, respectively. The structures of the modified nucleobases are shown inset. (**F** and **G**) SAM titrated into SAM-52 with 4-thiouridine substitutions at U9 and U20, respectively. (**H**) SAM titrated into SAM-49, derived from SAM-52 by deletion of three 3′-terminal nucleotides (see Supplementary Table S1). Nucleotide numbering was adjusted to correspond to that used for the *met*Y SAM-V riboswitch. ITC data for the *met*Y SAM-V riboswitch and variants are shown in Supplementary Figure S1.

Using isothermal titration calorimetry (ITC) we have studied ligand binding to two versions of the SAM-V riboswitch that control different genes, that were identified by Poiata *et al*. (13). These are the *met*Y and the *bhm*T riboswitches. The sequences are shown in Supplementary Table S1. The bulk of these analyses were performed using riboswitches of 55 and 52 nt in length, respectively. In these experiments ligand was titrated into 15 μM RNA and the heat changes measured (Figure 1 and Supplementary Figure S1). Titration of SAM into the *met*Y and the *bhm*T riboswitches resulted in the observation of exothermic binding, corresponding to the binding of one SAM molecule to the riboswitch with an affinities of 4.4 and 0.5 μM (Figure 1A; Supplementary Figure S1A and Table S2). By contrast, titration of *S*-adenosylhomocysteine (SAH) into either riboswitch led to no measurable evolution of heat, i.e. no detectable binding (Figure 1B and Supplementary Figure S1B). This confirms that the SAM-V riboswitches are highly selective for SAM, excluding the very similar SAH molecule. We have also analyzed variants of both riboswitches generated by atomic mutagenesis; these will be discussed below at the relevant points within the presentation of the structural data. Versions of the *met*Y and the *bhm*T riboswitches shortened by removal of 2 or 3 nt, respectively, from the 3′ terminus were also studied, and will be discussed below.

### Construction of *met*Y SAM-V riboswitch and crystallography

**Figure 2.**
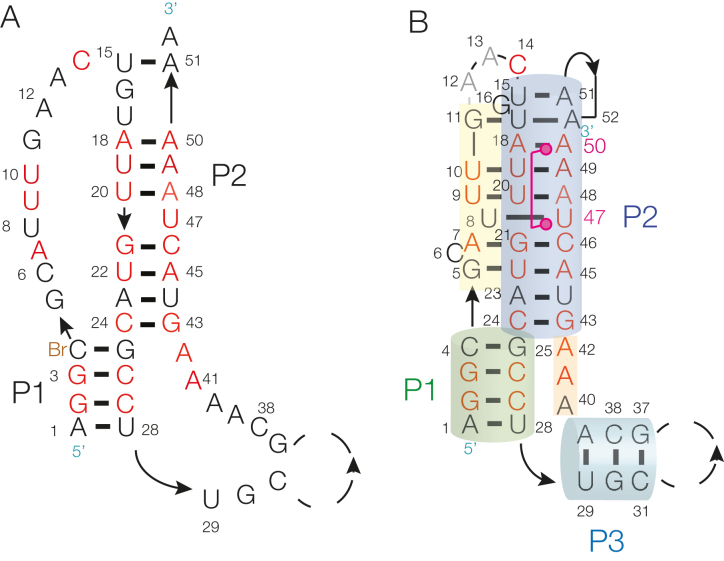
The sequence and secondary structure of the *met*Y SAM-V riboswitch studied by X-ray crystallography. (**A**) The sequence of the *met*Y SAM-V riboswitch with nucleotides that are highly conserved colored red. (**B**) The sequence of the *met*Y SAM-V riboswitch drawn to reflect the secondary structure observed by crystallography. The coloring scheme is used in the subsequent molecular graphics figures.

A 53-nt RNA corresponding to the *met*Y SAM-V riboswitch from *Candidatus* Pelagibacter ubique (13) (Figure 2A and Supplementary Table S1) was made by chemical synthesis. This included one 5-bromocytosine nucleotide at position 4 that was used to provide phase information by means of its anomalous scattering. The RNA was co-crystallized with SAM, in space group P4_1_22. The crystals diffracted to 2.5 Å resolution, and the structure was solved by single-wavelength anomalous diffraction (PDB ID: 6FZ0). The unit cell contained a single SAM-V molecule, and the quality of the electron density map was high, as can be judged from the experimental electron density shown in Figures 4–6. Details of data collection and refinement statistics for all the crystallographic data as deposited with the PDB are shown in Supplementary Table S3.

### The overall fold of the SAM-V riboswitch

**Figure 3.**
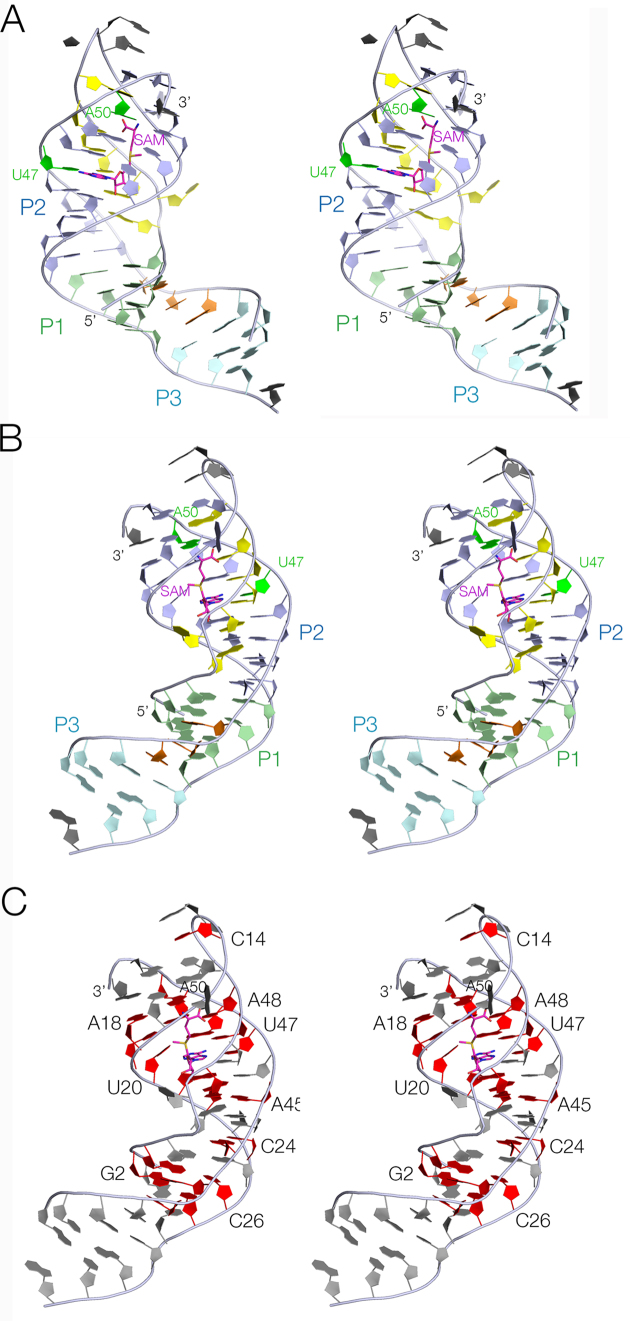
The overall structure of the *met*Y SAM-V riboswitch. Parallel-eye stereoscopic views of the structure are presented. (**A** and **B**) Views from opposite sides of the complete riboswitch structure. The SAM ligand is colored magenta. (**C**) A similar view to that in part B, with the conserved nucleotides colored red. Note the almost-complete conservation of the nucleotides surrounding the SAM binding site.

The base interactions are itemized in full in Supplementary Table S4. The core of the SAM-V riboswitch folds as an H-type pseudoknot, based upon two helical regions P1 and P2. Starting at the 5′ terminus (located at the bottom of the structure as depicted in Figures 2B and 3) the RNA forms the ascending strand of P1 with four base pairs, and then continues as the third strand in the major groove of P2 (G5–G11). After a turn comprising 3 nt (A12, A13 and C14) the strand forms the descending strand of P2, after which it continues directly as the descending strand of P1. From the 3′ end of P1 the strand forms a stem-loop based upon the 3 bp P3 helix. The base pairing of P3 is general, supported by the phylogenetic analysis summarized in Supplementary Figure S2. The loop is not visible in the electron density map, so is probably disordered within the crystal. Following P3, A40 to A42 form a continuous stack. A40, A41 and A42 face the minor groove of P1 where the nucleobases of the conserved A41 and A42 accept hydrogen bonds from O2′ of C27 and G3 respectively in A-minor interactions (specified in Supplementary Table S4). The strand then continues as the ascending strand of P2. The final nucleotide (A52) loops around to interact with U17 in the P2 helix; this is probably an artefact of the construct used and may not occur in the functional riboswitch (see ‘Discussion’ section). Supplementary Figure S3 shows a superposition of the pseudoknot cores of the SAM-V and *met*X SAM-II riboswitches. The structures are closely similar (RMSD = 0.373 Å) as anticipated from comparison of their secondary structures.

### The P2 triple helix

**Figure 4.**
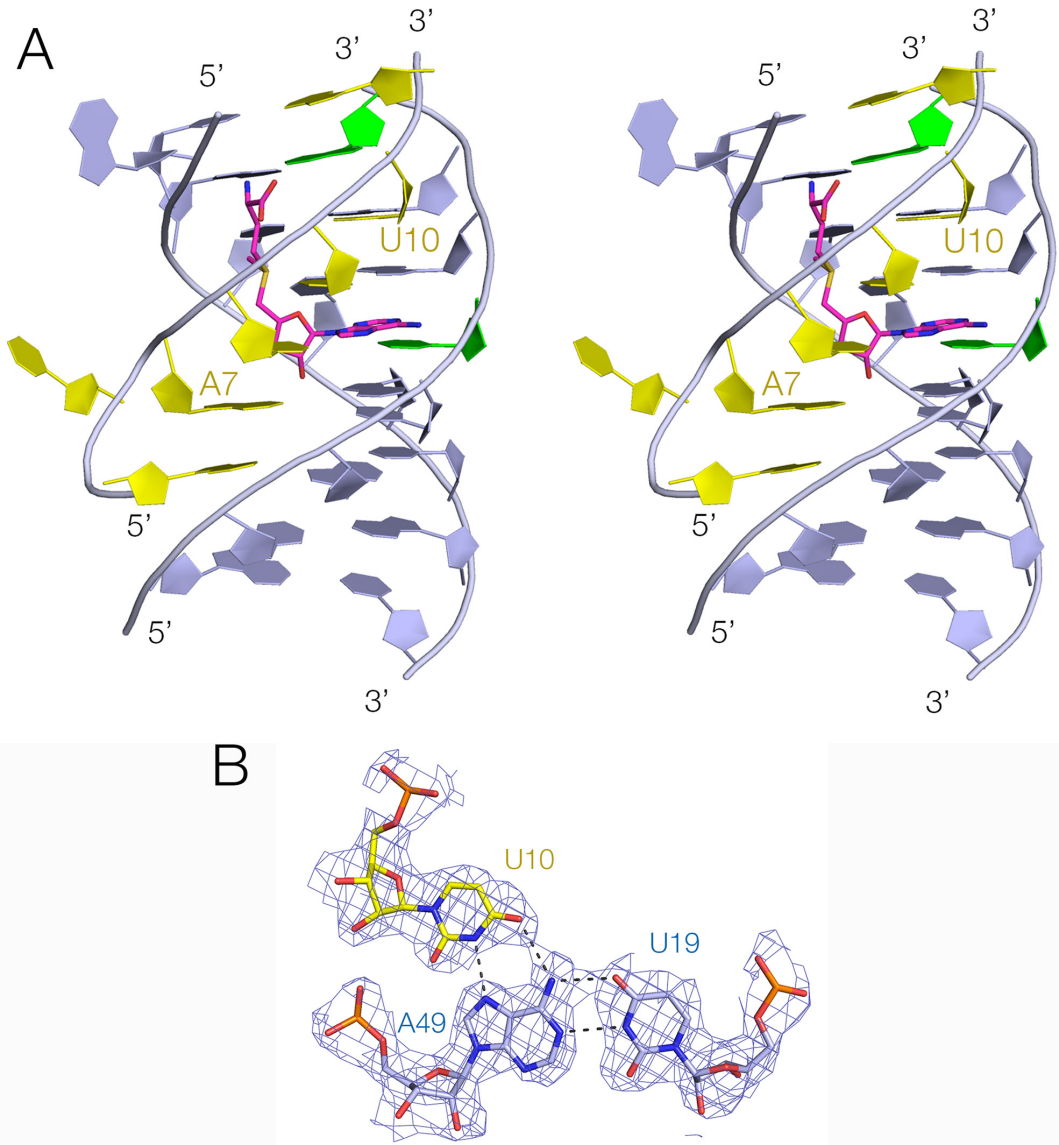
The P2 triple helix. (**A**) A parallel-eye stereoscopic view of the triple helix and the bound SAM ligand. The P2 duplex is colored blue and the third strand yellow. (**B**) A representative triple base interaction within the triplex. The U19:A49.U10 triple is shown with electron density from the experimental map contoured at 1.2 σ.

While P1 and P3 are standard duplex structures with *cis*-Watson–Crick base pairs, much of P2 forms an irregular triplex structure (Figure 4A). The third strand is located within the major groove of P2 and six (from a total of seven) of its nucleotides are hydrogen bonded to P2 nucleobases. Five of these form base triples of various kinds. All are *cis* Watson–Crick base pairs (derived from P2) with the third-strand nucleobase hydrogen bonded to the Hoogsteen edge. For example, two of the base triples are U:A.U, with the third strand U forming two hydrogen bonds with O6 and N7 of the adenine (Figure 4B). The 3′ end of P2 (at the top of the riboswitch as drawn) terminates with a *trans* Hoogsteen U:A basepair, while the lower end comprises two standard *cis* Watson–Crick basepairs before passing into the P1 helix. Most the nucleotides comprising the triplex region are >97% conserved (Figure 3C and Supplementary Figure S2) (13), and this section of the riboswitch forms the ligand binding site. A full list of all the base interactions are presented in Supplementary Table S4.

### SAM binding by the riboswitch

**Figure 5.**
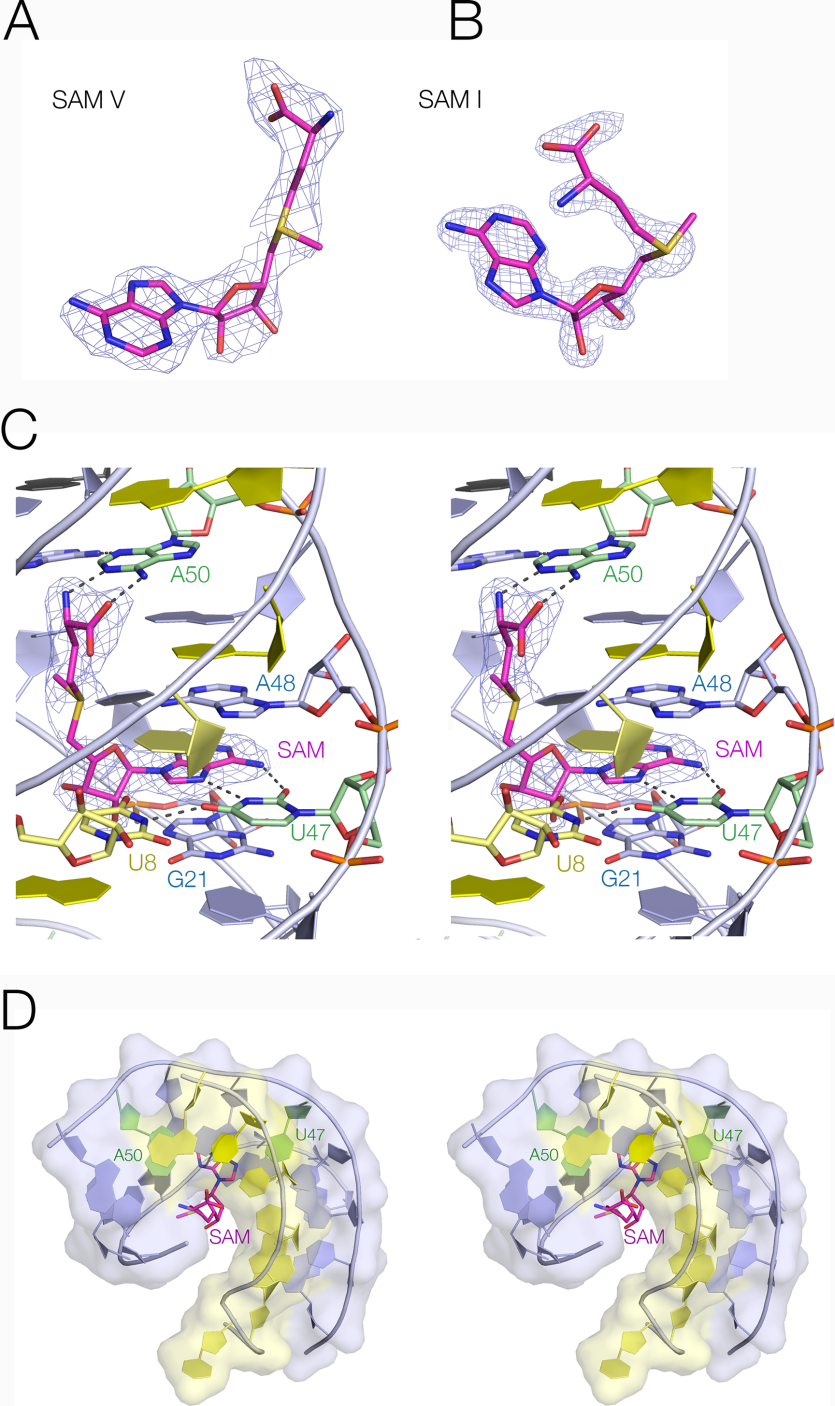
The SAM binding site. (**A** and **B**) The conformation of SAM in the *met*Y SAM-V riboswitch and the SAM-I riboswitch, respectively. The latter was taken from PDB ID: 5FJC at 1.71 Å resolution (32). (**C**) A parallel-eye stereoscopic view of the SAM binding site, with electron density from the experimental map contoured at 1.2 σ shown on the SAM ligand. (**D**) A parallel-eye stereoscopic view down the axis of the P2 triplex showing position of the bound SAM ligand.

The chain of the SAM ligand bound to the riboswitch is maximally extended and aligned with the helical axis. The adenine moiety has a C3′-*endo* pucker with an *anti* glycosyl conformation (Figure 5A). The SAM is hydrogen bonded to the RNA at both ends, with no direct contacts with the remaining methionyl chain (Figure 5C).

**Figure 6.**
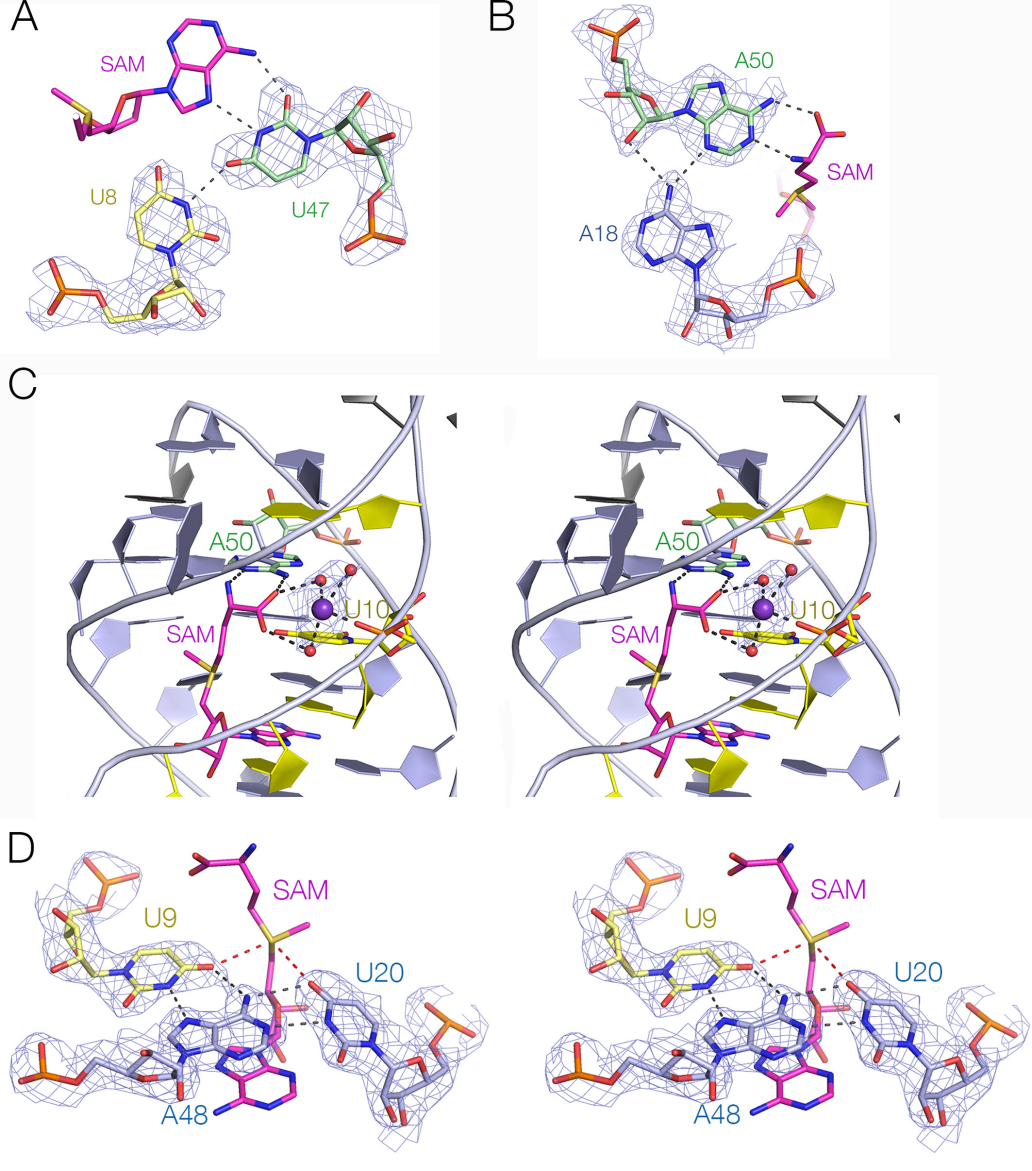
The interactions binding SAM to the SAM-V riboswitch. (**A**) The interaction of the SAM adenine moiety with U47. (**B**) The interaction of the SAM amino acyl end interaction with A50. In parts A and B the electron density from the experimental map contoured at 1.2 σ shown for the RNA. (**C**) A metal (most probably Mg^2+^) ion bound to the SAM carboxyl and the P2 backbone. There is a direct metal-oxygen bond to the *pro*-R phosphate oxygen atom of U10. The metal ion is colored purple and water molecules of hydration red. The electron density shown on the hydrated metal ion is taken from the experimental map contoured at 1.5 σ. (**D**) The U20:A48.U9 base triple half-surrounding the SAM sulfonium. While there is no direct contact, the O4 atoms of U20 and U9 are only 3.2 Å from the sulfonium cation. The red broken lines highlight the proximity of the O4 and S atoms. The electrostatic interaction should be a significant factor in the discrimination between SAM and the neutral SAH. The electron density shown on the base triple is taken from the experimental map contoured at 1.2 σ.

The adenine nucleobase of SAM is basepaired with U47 as a *trans* Hoogsteen basepair, with hydrogen bonds from N6 to U47O2 and from U47N3 to N7. U47 is also hydrogen bonded to U8 of the third strand, donated by U8N3 to U47O4 (Figure 6A). The adenine nucleobase is intercalated into the P2 helix, stacked in between those of G21 and A48, and is thus flanked by base triples on both sides. Substitution of U47 by isocytidine (that should preserve the base pair with U8 but will disrupt both hydrogen bonds to the SAM adenine moiety) completely prevented detectable binding of SAM to either the *met*Y and the *bhm*T riboswitches (Figure 1C and Supplementary Figure S1C).

At the other end of the SAM chain the aminoacyl group is hydrogen bonded to the nucleobase of A50, donating a proton from N6 to the carboxyl O and accepting one at N1 from the amine (Figure 6B). A50 is hydrogen bonded to A18, accepting protons from A18N6 at N3 and O2′, although the latter is somewhat out of plane. The A50:A18 pair is stacked between base triples on both sides. The carboxyl group makes additional contacts to a hydrated metal ion that is bound site-specifically to the RNA (Figure 6C); the metal-O distance is 2.1 Å, so this is most likely a Mg^2+^ ion. The metal ion is bonded directly to the *pro*R non-bridging phosphate oxygen atom of U10, while both SAM carboxylate O atoms are hydrogen bonded to inner-sphere water molecules. Substitution of A50 by purine (i.e. loss of 6 NH_2_) will remove the hydrogen bond to the SAM carboxyl while retaining that to the amine. This weakened but did not prevent SAM binding in the *met*Y and the *bhm*T riboswitches (Figure 1D; Supplementary Figure S1D and Table S2). Substitution by inosine (i.e. conversion of 6 NH_2_ to O) would remove both hydrogen bonds to the amino acid; this led to severe loss of binding in both riboswitches (Figure 1E; Supplementary Figure S1E and Table S2). Note that neither substitution should prevent formation of the A50:A18 basepair.

We note that the nucleotides that are hydrogen bonded to the ligand (U47 and A50) both form base pairs that appear to be relatively weak and are therefore likely to be stabilized by the interaction with the ligand, so that the structure of P2 overall is stabilized when SAM is bound. This is probably important in the function of the riboswitch.

The SAM sulfonium is coplanar with the U20:A48.U9 base triple, lying on the helical axis and thus surrounded by the triple on one side. The positively charged sulfur atom is 3.2 and 3.3 Å from the O4 carbonyl atoms of U20 and U9 (Figure 6D). Quantum mechanical calculations indicate that the C-O4 bond of uracil has a dipole moment that generates a significant negative electric charge at O4 (24), so in a region of relatively low dielectric there could be a significant stabilization of the bound SAM by electrostatic interaction. By contrast, binding of the electrically-neutral *S*-adenosyl homocysteine would not be stabilized in this manner. Substitution of U9 by 4-thiouridine (that will lower the partial charge on the exocyclic atom at the 4 position) completely prevented detectable binding of SAM to the *bhm*T riboswitch (Figure 1F). Corresponding substitution of U20 by 4-thiouridine severely reduced binding of SAM (Figure 1G).

The conformation of the bound SAM and its hydrogen bonding with the RNA is very similar in both the SAM-V and *met*X SAM-II (11) riboswitches. This supports the close structural similarity of the two riboswitches that is suggested by their proposed secondary structures.

### Importance of the 3′-terminus of the SAM-V riboswitch for ligand binding

Loss of 2 nt from the 3′-terminus of the *met*Y riboswitch (i.e. the form for which the structure was solved by crystallography) prevented any binding of SAM detectable by calorimetry (Supplementary Figure S1F). The analogous experiment was performed using the *bhm*T riboswitch by deletion of three nucleotides from the 3′-terminus (Figure 1H). Again no binding of SAM could be detected. Analysis of these two SAM-V riboswitches demonstrates the critical importance of this 3′ sequence, and we discuss the probable role of this in the function of the riboswitch below.

## DISCUSSION

As a class, riboswitches alter their conformation in response to the binding of a ligand to regulate gene expression. To achieve this they must bind their principal ligand with high selectivity, excluding very similar species. The SAM-V riboswitch binds its ligand SAM with micromolar affinity, and excludes SAH. SAH differs from SAM only in the absence of the methyl group, and thus a positive charge, on the sulfur atom. The affinity for SAM is >1000-fold greater than for SAH (13), corresponding to a difference in binding free energy of >17 kJ mol^−1^.

### Ligand binding by the SAM-V riboswitch, and electrostatic discrimination of SAM and SAH

The SAM V riboswitch with bound ligand folds as an H-type pseudoknot structure (Figures 2 and 3) in which the third strand locates in the major groove of the 3′ helix (P2) to form a triple helix (Figure 4). This creates the SAM binding site, located in the major groove of P2 (Figure 5C). The SAM is held by hydrogen bonding at the two extremes. At one end the adenine is stacked into the helix and forms a *trans* Hoogsteen base pair with U47. At the other end of SAM the aminoacyl group is hydrogen bonded to A50, and the carboxylate is hydrogen bonded to inner-sphere water molecules of a metal ion that is directly bound to the RNA backbone. In between these two anchor points the SAM chain is extended along the axis (Figure 5D), making no direct contacts. This arrangement fixes the sulfonium cation so that it is surrounded on one side by the U20:A48.U9 triple, directing the two uridine C-O4 vectors toward the sulfur with O-S distances of ≤ 3.3 Å (Figure 6D). The O4 atoms carry a significant negative charge, and in a region of low dielectric the electrostatic interaction with the sulfonium will be expected to be substantial. Substitution of either uracil O4 by sulfur (that has significantly lower electronegativity than oxygen) resulted in little or no binding of SAM (Figure 1F and G). Since the sulfur atom of SAH is uncharged there will be no electrostatic stabilization of this compound, and the loss of the electrostatic energy is probably a major contributor to the discrimination between SAM and SAH.

### Modulation of translational initiation by the SAM-V riboswitch

The formation of the P2 triplex creates a binding site for SAM; conversely the binding of SAM should stabilize the P2 triplex. U47 and A50 that are the anchor points at the two extremes of SAM are both involved in weak base pairing, with U8 and A18, respectively; these nucleobases are each connected by single hydrogen bonds and thus likely to be stabilized by interaction with SAM. The electrostatic interaction between the SAM sulfonium cation should stabilize the U20:A48.U9 triple, and the carboxylate of SAM is hydrogen bonded to a metal ion that is bound to the backbone of P2. It is therefore probable that in the absence of bound SAM the third strand is released from the major groove, leaving P2 as a simple duplex with regular Watson–Crick base pairing between U20:A48 and C24:G43 inclusively and U47 as a single-nucleotide bulge. This leaves the 3′ end of the RNA that contains the Shine-Dalgarno sequence free to interact with the 30S ribosomal subunit to initiate translation of the downstream gene. This would therefore represent the ON state of the riboswitch.

Binding the SAM ligand is then expected to stabilize the triplex form of P2 as discussed. Our calorimetric measurements show the importance of the 3′ sequences of the *met*Y and *bhm*T SAM-V riboswitches (Figure 1H and Supplementary Figure S1F), and it is probable that for these longer sequences there is no base pairing between A52 and U17. Rather, we note that alignment of SAM-V sequences (Supplementary Figure S2) reveals complementarity between the sequence linking the P2 third strand and the descending strand of the P2 duplex, i.e. nt 14 and 15 in our numbering scheme. Thus a short additional helix forms immediately 3′ to the riboswitch (P2a), and this includes part of the Shine-Dalgarno sequence that begins at A52 (Supplementary Table S5). A 2-bp helix is indeed observed in the structure of the metX SAM-II riboswitch (11); this is a rare translational SAM-II riboswitch. The binding of SAM thus folds the SAM-V riboswitch into a structure that occludes the sequence that is necessary to initiate ribosome binding, i.e. this is the OFF state of the riboswitch.

**Figure 7.**
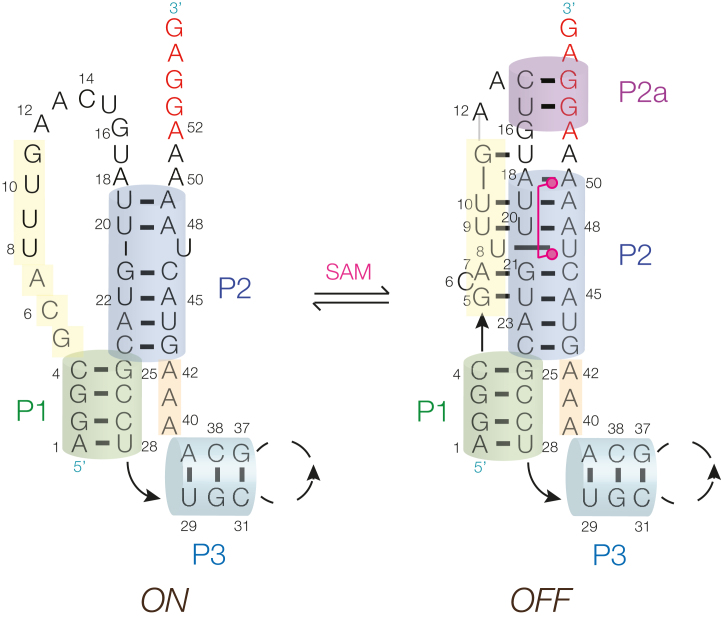
A model for the function of SAM-V riboswitch. We propose that in the absence of bound SAM, while the P1 and P2 duplexes are base paired, the third strand of P2 (yellow) is disengaged and the P2a helix is not formed. This allows the Shine-Dalgarno sequence (red) to bind the ribosome to initiate translation. This is the ON state of the riboswitch. On binding SAM the third strand is stably bound to P2 as a major-groove triplex, and the P2a helix is formed so occluding the ribosome binding site. Translation is not initiated, and thus this is the OFF state of the riboswitch.

The proposed mechanism of the riboswitch is summarized by the model shown in Figure 7. In the absence of bound ligand the third strand of P2 is disengaged, the P2a helix is not formed and the Shine-Dalgarno accessible to the ribosome. Upon binding SAM the P2 triplex form is stabilized, P2a forms and prevents the ribosome from binding to the Shine-Dalgarno sequence. This is closely similar to the mechanism proposed for the metX SAM-II riboswitch (11).

The model we propose for the SAM-V riboswitch is broadly in agreement with the SAM-induced protection observed by Poiata *et al*. (13) in in-line probing experiments. The strands of P2 and P3 are essentially protected (i.e. structured) without addition of SAM suggesting that these duplexes are formed irrespective of the presence of ligand. By contrast the main change occurs in the section that forms the third strand of P2, consistent with SAM-induced triplex formation. Thus addition of SAM led to reduced reactivity of the third strand at G5, U16, U17 and U18 that forms the triplex. However there was a marked increase in reactivity of C6 (C14 in the numbering scheme of Poiata *et al*. (13)) that is bulged out in the triplex form. Our crystal structure explains this hyper-reactivity. While the position of the C6 nucleobase is not defined (it is probably flexible), the electron density for the ribose and particularly the O2′ are clearly observed (Supplementary Figure S4). The O2′ is 3.3 Å from the adjacent P, and the O2′-P-O5′ angle is 157°, corresponding to an ‘in-line fitness’ parameter (25) *F* = 0.69 so that the structure is fully consistent with the observed reactivity. In addition, the region that forms P2a in the model also exhibit reduced reactivity in the presence of SAM. Taken together the in-line probing data strongly support the model represented in Figure 7.

### Comparison of SAM-V with other SAM-binding riboswitches

It is interesting to compare the structure and ligand binding of the SAM-V riboswitch with the other SAM riboswitches, particularly SAM-I, II and III (7,9,10) for which crystal structures are available (8,11,12). A clear similarity exists between the SAM-V and SAM-II riboswitches, and in their original paper describing the SAM-V riboswitch, Poiata *et al*. (13) noted a strong similarity in secondary structure between the two riboswitches. Both sequences suggested the formation of a pseudoknot. However, the two riboswitches have a different bacterial distribution, and the majority of the SAM-II riboswitches are transcriptional whereas the SAM-V riboswitch is translational. A major difference appeared to be that an additional 3′ helix P2a was proposed for the SAM-II riboswitch. However, we now find that the structures of SAM-II (11) and SAM-V are closely similar, and their manner of binding the SAM ligand virtually identical (11,26). Furthermore, the core of the riboswitches where the SAM ligand binds can be almost perfectly superimposed in the two structures (Supplementary Figure S3). A comparison of the secondary structures of the SAM-II and SAM-V riboswitches and their phylogenetic distributions are shown in Supplementary Figure S5. The lengths of the P1 and P2 helices are different in the two riboswitches, and P3 is absent in the SAM-II riboswitch. Importantly, the P2a helices in 60% of the SAM-II riboswitches are 5 bp in length, compared to just 2 bp in the SAM-V riboswitch and tend to comprise C-G base pairs. Thus the SAM-II riboswitches have a stable P2a helix, whereas that of the SAM-II riboswitch is likely to be much less stable and so require the binding of SAM ligand for stabilization. The P2a helix includes the probable Shine-Dalgarno sequence in at least 86% of the SAM-V riboswitches (Supplementary Table S5). Thus the likely chief functional difference between the SAM-II and SAM-V riboswitch is that the former tend to have longer, stable P2a helices that should exist with or without bound ligand, whereas that of the SAM-V riboswitch is probably bistable. In the case of the SAM-II riboswitches the association of the P2a helix with a Shine-Dalgarno sequence is not obvious, consistent with a role in transcriptional regulation rather than translational. A minor sub-set of SAM-II riboswitches are translational, including metX for which the crystal structure was solved by Batey and co-workers (11), and in that case the P2a helix is just 2 bp in length i.e. the same as that of the SAM-V riboswitch. Because all recent experimental and computational studies of the SAM-II riboswitch have focused on the metX SAM-II sequence (27–29) these also potentially provide insight into the function of the SAM-V riboswitch. A computational study by Sanbonmatsu and co-workers (29) based on the structure of the metX SAM-II riboswitch concluded that the binding of the SAM ligand stabilizes both the triplex and P2a, closely similar to the model we propose here for the SAM-V riboswitch.

By contrast, SAM-V and II differ fundamentally from the SAM-I and III riboswitches. The latter are based on helical junctions (four- and three-way, respectively) whereas the former both fold as H-type pseudoknots. It is interesting to note that the recently described structure of the guanindine-III riboswitch (30) is also an H-type pseudoknot, and ligand binding occurs as an integral part of the major-groove triple helix that forms. But the guanindine-III riboswitch is a translational ON switch (31), so ligand-induced folding of the RNA must release the ribosome binding site for interaction with the ribosomal small subunit. The manner of SAM binding by the SAM-I and III riboswitches is also markedly different from SAM-V and II. The conformation of bound SAM in SAM-V and I riboswitches is shown in Figure 5A and B. While the SAM chain of the SAM-V riboswitch is maximally extended, with an end-to-end distance of 11.8 Å, that of the SAM-I riboswitch bends by 90° at the sulfur atom, shortening the end-to-end distance to 5.8 Å. Furthermore the SAM adenine adopts a *syn* conformation in the SAM-I riboswitch. The adenine is base paired via its Hoogsteen edge, and the aminoacyl group is hydrogen bonded to two nucleobases, but there is no equivalent of the U20:A48.U9 triple that might make an electrostatic interaction with the sulfonium in a similar manner.

## CONCLUSION

The structure of the SAM-V riboswitch indicates how it meets the twin objectives of ligand specificity and ligand-dependent regulation of gene expression. The RNA folds into a pseudoknot structure that includes a major-groove triple helix that creates a pocket that is highly specific for its SAM ligand. The SAM is hydrogen bonded at the adenine and amino acid ends, and the connecting chain is fully extended along the axis of the triple helix. This suspends the sulfonium ion coplanar with a U:A.U base triple such that both uridine O4 electronegative atoms are directed toward the cation. This explains how the riboswitch is selective for SAM, since the neutral SAH will not be subject to the electrostatic stabilization. The Shine-Dalgarno ribosome binding site is located immediately 3′ to the riboswitch and phylogenetic evidence suggests this should form a short helix P2a, preventing ribosomal access and thus downregulating translation—the OFF state. In the absence of SAM it is probable that the third strand disengages from the P2 triplex, destabilizing helix P2a and switching the riboswitch to its ON state where the ribosome can bind to initiate translation of the downstream gene.

## DATA AVAILABILITY

The structure was deposited in the PDB under ID 6FZ0.

## Supplementary Material

Supplementary Data
