# Supplementary material for: Structure and ligand binding of the SAM-V riboswitch
Source: Nucleic Acids Res. 2018 Jun 21;46(13):6869–79. doi: 10.1093/nar/gky520 (PMC6061858; doi:10.1093/nar/gky520)
Supplement: Supplementary Data [file gky520_supplemental_files.pdf]

Structure and ligand binding of the SAM-V riboswitch

Lin Huang and David M. J. Lilley

SUPPLEMENTARY MATERIALS

Comprising five supplementary figures and five supplementary tables.

SUPPLEMENTARY FIGURES

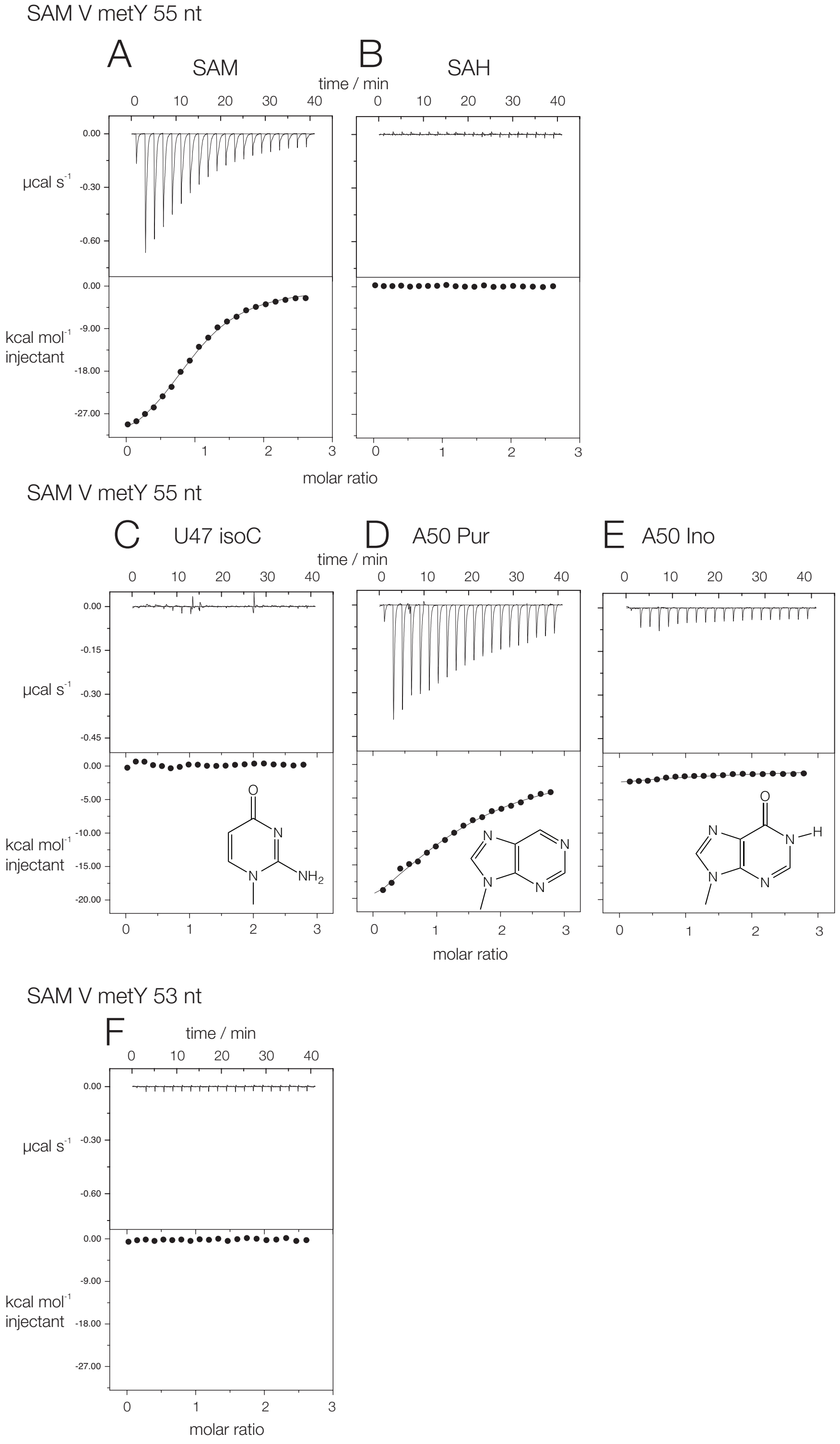

Figure S1. Isothermal titration calorimetric measurement of ligand binding to the *met*Y SAM-V riboswitch. In all titrations except **F** the RNA was the 55 nt form of the riboswitch, and all except part **B** were titrated with SAM. Titrations **A** and **B** used unmodified riboswitch, and In **B** SAH was titrated into the RNA. In parts **C**, **D** and **E**, SAM was titrated into the SAM-V variants U47 isoC (isocytosine), U50Pur (purine) and U50Ino (inosine) respectively. A form of *met*Y SAM-V deleted in the two 3’ nucleotides (see Table S1) was used in part **F**. Thermodynamic data are shown in Table S2.

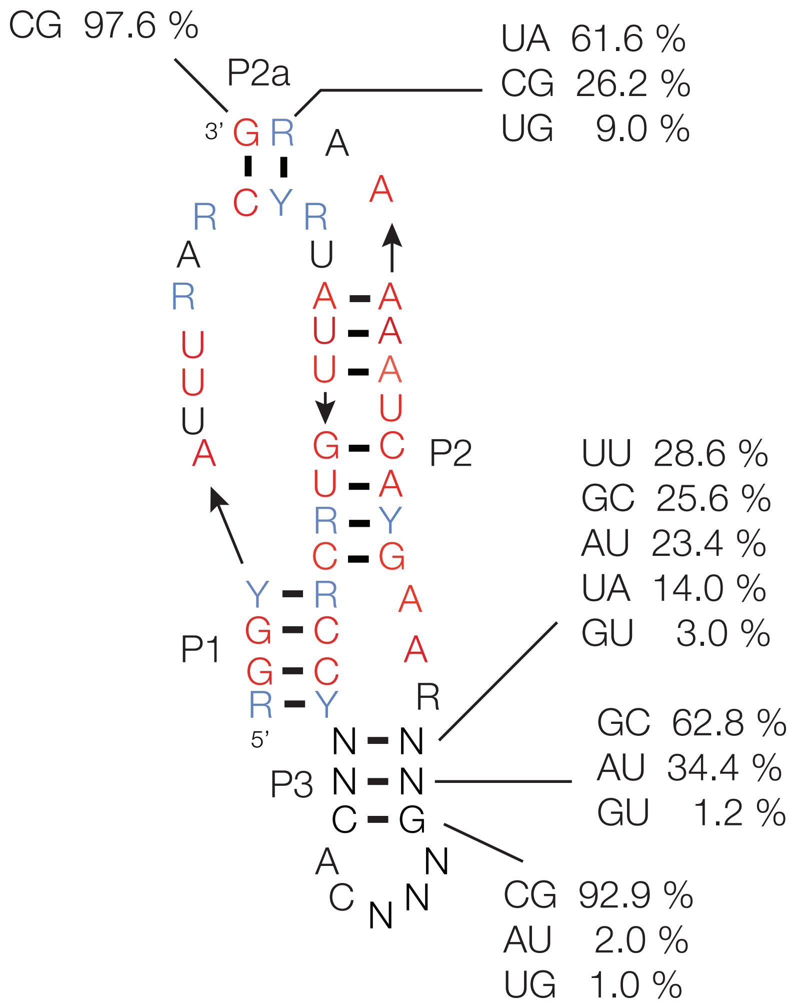

Figure S2. Conservation of nucleotides in the SAM-V riboswitch. Nucleotides conserved >97% are shown in red, and those conserved as either purine (R) or pyrimidine (Y) are shown blue. The conservation of each base pair type in helices P3 (observed in the crystal structure) or P2a proposed here are shown. Base pairs of frequency <1 % are not shown.

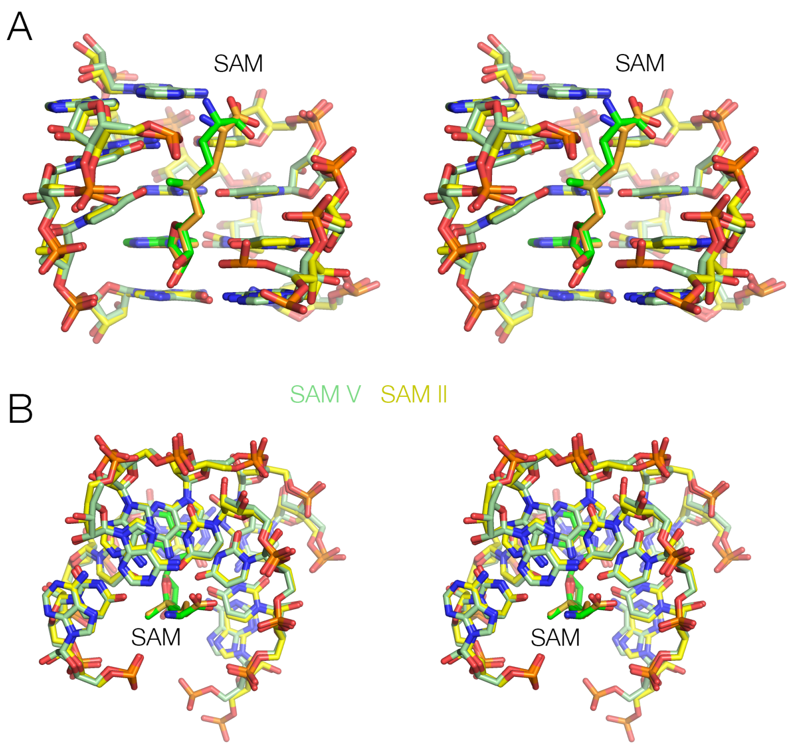

Figure S3. Comparison of the structures of the SAM-binding cores of the SAM V (green) and *met*X SAM II (PDB ID 2QWY) (yellow) riboswitches. The cores have been superimposed (RMSD = 0.373 Å) and two parallel-eye stereoscopic views are shown. **A**. Side view showing the binding site of the SAM. **B**. View down the axis of the triple helical region.

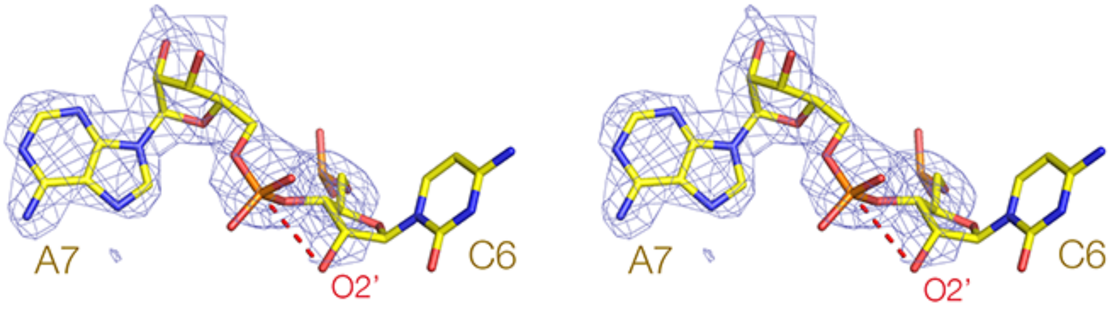

Figure S4. The local geometry at the phosphate group connecting C6 and A7 in the *met*Y SAM-V riboswitch. A parallel-eye stereoscopic view of the dinucleotide showing the experimental phasing map. The electron density for the C6 nucleobase is weak, indicative of mobility, but that for the ribose is clear, showing the position of the O2’ atom. This is 3.3 Å from the adjacent phosphate, with a O5’-P-O2’ angle of 157°. These values correspond to a very high “in-line fitness” parameter. The broken red line shows the direction of attack by the O2’ that would lead to backbone scission at that point in in-line probing experiments just as observed experimentally.

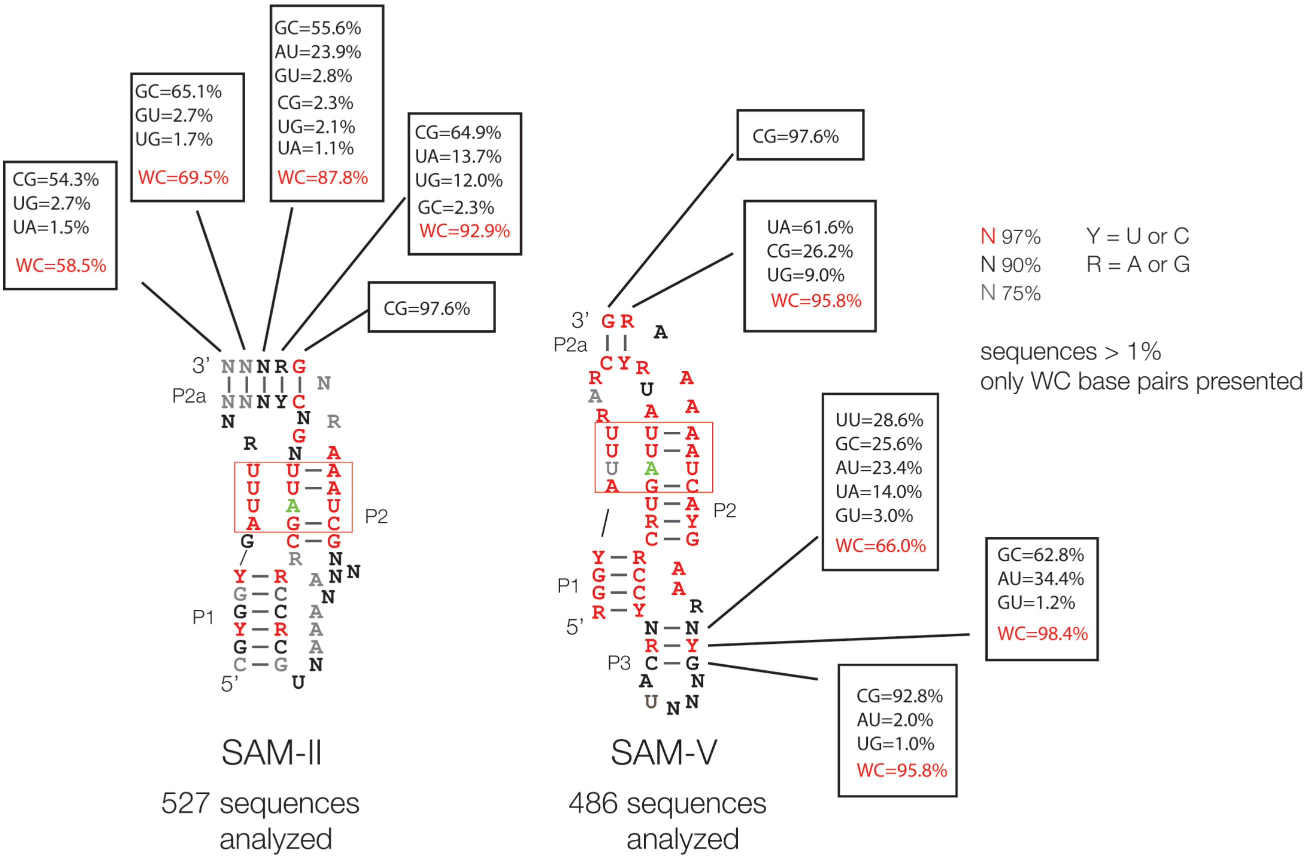

Figure S5. Comparison of the secondary structures of the SAM-II and SAM-V riboswitches. The conserved cores of the two riboswitches are boxed. Phylogenetic analysis indicates the following general points :

1. The P1 helix of the SAM-II riboswitch is generally ≥ 6 bp (usually 6 bp). In the SAM-V riboswitch P1 is 4 bp.
2. The P2 helix of the SAM-II riboswitch is always 4 bp, but in the SAM-V riboswitch P2 is 6 bp.
3. The majority of SAM-V riboswitches have a P3 helix, whereas the SAM-II riboswitches do not.
4. The 3' termini of the SAM-V riboswitches have obvious Shine-Dalgarno sequences in 85 % of cases, see Table S5. Those of the SAM-II riboswitches are less conserved, and so less clear.

The A written green is the adenine of the SAM ligand. In the phylogenetic analysis only Watson-Crick basepairs are shown, and only those sequences with a frequency > 1% are listed.

SUPPLEMENTARY TABLES

metY 53 nt (used for crystallization) :

AGGCGCAUUUGAACUGUAUUGUACGCCUUGCAUAAAGCAAAAGUACUAAAAAA

metY 55 nt :

AGGCGCAUUUGAACUGUAUUGUACGCCUUGCAUAAAGCAAAAGUACUAAAAAAGG

*bhm*T 49 nt :

GGGCAGUUGAACCAUAUUGUGCGCCCUGCAUUUGCUUAAGCACUAAAAA

*bhm*T 52 nt :

GGGCAGUUGAACCAUAUUGUGCGCCCUGCAUUUGCUUAAGCACUAAAAAGGA

Table S1. Sequences of *met*Y and *bhm*T SAM-V riboswitches used in these studies. All sequences are written 5' to 3'. Two lengths of each have been studied, with 2 or 3 nt 3' extensions respectively. The 53 nt version of the *met*Y riboswitch was crystallized, with bromocytidine incorporated at the nucleotide highlighted in red.

Name	length nt	n	ΔH / kcal.mol^{-1}	ΔS / cal.K^{-1}mol^{-1}	ΔG / kcal.mol^{-1}	K_d / μM
*met*Y	55	0.99 ± 0.01	-36.2 ± 0.5	-96.9	-7.31	4.37 ± 0.21
*met*Y A50Pur	55	1.36 ± 0.07	-33.2 ± 2.6	-89.2	-6.59	14.7 ± 1.8
*bhm*T	52	0.94 ± 0.01	-37.3 ± 0.7	-96.3	-8.60	0.50 ± 0.08
*bhm*T A50Pur	52	0.69 ± 0.01	-41.3 ± 1.0	-111	-8.25	0.90 ± 0.10
*bhm*T A50Ino	52	1.11 ± 0.11	-14.7 ± 2.1	-27.6	-6.51	16.8 ± 2.6

Table S2. Thermodynamic parameters for SAM binding to *met*Y and *bhm*T SAM-V riboswitches and some variants generated by atomic mutagenesis. Several of the atomic mutants lead to no measurable evolution of heat on titration of SAM, so that no parameters can be obtained. These are not therefore included in this table.

ligands	SAM
PDB	6FZ0
**Data collection**	
Space group	P $4_1$22
Cell dimensions	
a, *b*, *c* (Å)	87.8, 87.8, 62.7
α, *β*, *γ* (°)	90 90 90
	SAD-Br
	Peak
Wavelength	0.9196
Resolution (Å)	44.11– 2.50 (2.54 – 2.50)
R_{merge}	0.083 (0.976)
I / σ*I*	16.8 (2.1)
CC (1/2)	1.00 (0.38)
Completeness (%)	100 (99.3)
Redundancy	13.8 (13.2)
**Refinement**	
Resolution (Å)	44.11– 2.50 (2.66 – 2.50)
No. reflections	15977 (2562)
R_{work} / R_{free}	0.234 / 0.270
No. atoms	
macromolecules	1018
ligands	30
B-factors	
macromolecules	92.05
ligands	86.96
solvent	59.85
R.m.s. deviations	
bond lengths (Å)	0.002
bond angles (°)	0.60

Table S3. Details of data collection and refinement statistics for the crystallographic data as deposited with the PDB.

*Values in parentheses are for highest-resolution shell.

Nucleotide	Nucleotide	Interactions
A1	U28	P1 *cis*-WC
G2	C27	P1 *cis*-WC
G3	C26	P1 *cis*-WC
O2'	A42 N1	P1 minor groove interaction
C4	G25	P1 *cis*-WC
G5 N1 O6	A45 N7 N6	*cis*-WC-H; A45:U22.G5 triple
C6	-	extruded
A7 N6	G21 O6	*cis*-WC
N1	C46 N4	*trans*-H; C46:G21.A7 triple
U8 N3	U47 O4	*cis*-WC; SAM adenine binding
U9 N3 O4	A48 N7 N6	*cis*-H; A48:U20.U9 triple
U10 N3 O4	A49 N7 N6	*cis*-WC-H; A49:U19.U10 triple
G11 N1	U17 O4	*trans*-WC-H; A52:U17.G11 triple
A12		turn
A13		turn
C14		turn
U15 O2 N3	A51 N6 N7	*trans*-WC-H ; truncation artefact
G16	-	extruded
U17	G11	*trans*-H-WC; A52:U17.G11 triple
O2 N3	A52 N6 N1	P2 t*rans*-WC; A52:U17.G11 triple
A18 N6	A50 N3	P2 t*rans*-H-S; SAM aminoacyl binding
U19 N3 O4	A49 N1 N6	P2 *cis*-WC; A49:U19.U10 triple
U20 N3 O4	A48 N1 N6	P2 *cis*-WC; A48:U20.U9 triple
G21 O6	A7 N6	*trans*-H; C46:G21.A7 triple
N1 O6 N2	C46 N3 N4 O2	P2 *cis*-WC; C46:G21.A7 triple
U22 N3 O4	A45 N1 N6	P2 *cis*-WC; A45:U22.G5 triple
A23	U44	P2 *cis*-WC
C24	G43	P2 *cis*-WC
G25	C4	P1 *cis*-WC
C26	G3	P1 *cis*-WC

C27	G2	P1 *cis*-WC
O2’	A41 N3	P1 minor groove interation
U28	A1	P1 *cis*-WC
U29	A39	P3 *cis*-WC
G30	C38	P3 *cis*-WC
C31	G37	P3 *cis*-WC
G37	C31	P3 *cis*-WC
C38	G30	P3 *cis*-WC
A39	U29	P3 *cis*-WC
A40		linking
A41	C27	linking; P1 minor groove interaction
A42	G3	linking; P1 minor groove interaction
G43	C24	P2 *cis*-WC
U44	A23	P2 *cis*-WC
A45	G5	*cis* H-WC; A45:U22.G5 triple
	U22	P2 *cis*-WC; A45:U22.G5 triple
C46	A7	*cis*-WC; C46:G21.A7 triple
	G21	P2 *cis*-WC; C46:G21.A7 triple
U47	U8	*cis*-WC; SAM adenine binding
A48	U9	*cis*-H-WC; A48:U20.U9 triple
	U20	P2 *cis*-WC; A48:U20.U9 triple
A49	U10	*cis*-H-WC; ; A49:U19.U10 triple
	U19	P2 *cis*-WC; A49:U19.U10 triple
A50	A18	P2 t*rans*-S-H; SAM aminoacyl binding
A51	U15	*trans*-H-WC ; truncation artefact
A52	U17	P2 t*rans*-WC; A52:U17.G11 triple

Table S4. List of internucleotide contacts in the *met*Y SAM-V riboswitch structure. Basepairing : WC = Watson-Crick, H=Hoogsteen, S=sugar edge. Standard *cis*-WC base pairs hydrogen bonding not specified.

Sequence	number
AAGGAGU	143
AGGAGAA	117
AAGUGAGG	69
AAGGAGA	46
AGGAGAG	16
AGGAGGA	13
AGGAGAU	8
AGGAGUA	4
ACGGAGG	2
UAGGAGA	1
Total	419

Table S5. Shine-Dalgarno sequences in SAM-V riboswitches. The Shine-Dalgarno sequences are highlighted in blue type, and the sequences that should form P2a helices (supported by covariation analysis) are underlined. The 419 sequences listed here comprise 86.2 % of the total 486 sequences analyzed.
